# Proposal to set up a College of Family Medicine in East, Central and Southern Africa

**DOI:** 10.4102/phcfm.v14i1.3612

**Published:** 2022-09-05

**Authors:** Sunanda Ray, Farai D. Madzimbamuto

**Affiliations:** 1 Department of Medical Education, Faculty of Medicine, University of Botswana, Gaborone, Botswana; 2 Department of Anaesthetics and Critical Care, Faculty of Medicine, University of Botswana, Gaborone, Botswana

**Keywords:** Family medicine training, College of Family Medicine, East Africa, Central Africa, Southern Africa

## Abstract

Family Medicine training in Africa is constrained by limited postgraduate educational resources and opportunities. Specialist training programmes in surgery, anaesthetics, internal medicine, paediatrics and others have developed a range of trainers and assessors through colleges across East, Central and Southern Africa (ECSA). Each college has a single curriculum with standardised training and assessment in designated institutions, which run alongside and in collaboration with the Master’s in Medicine programmes in universities. Partnerships between colleges in Britain, Ireland and Canada and national specialist associations have led to joint training-of-trainer courses, e-learning platforms, improved regional coordination, better educational networking and research opportunities through regional conferences and joint publications. We propose the establishment of a regional college for specialist training of family physicians, similar to other specialist colleges in ECSA. Partnerships with family medicine programmes in South Africa, Canada and Australia, with support from international institutions such as the Primary Care and Family Medicine Network for Sub-Saharan Africa (PRIMAFAMED) and the World Organisation of Family Doctors (WONCA Africa), would be essential for its success. Improved health outcomes have been demonstrated with strong primary care systems and related to the number of family physicians in communities. A single regional college would make better use of resources available for training, assessment and accreditation and strengthen international and regional partnerships. Family medicine training in Africa could benefit from the experience of specialist colleges in the ECSA region to accelerate training of a critical mass of family physicians. This will raise the profile of family medicine in Africa and contribute to improved quality of primary care and clinical services in district hospitals.

## Background

The Second Africa Regional Meeting of the World Organization of Family Doctors (WONCA), in a consensus statement reflecting the growth of the discipline of Family Medicine in Africa, defined the role of the family physician in Africa as ‘a clinical leader and consultant in the primary health care team, ensuring primary, continuing, comprehensive, holistic and personalised care of high quality to individuals, families and communities’.^1^ This role was further endorsed at the Third Africa Regional Meeting of WONCA held at Victoria Falls on 19–21 November 2012, where the progress of many countries in Africa in establishing postgraduate training programmes in family medicine was acknowledged.^2^ Internationally, research shows that stronger primary care services are linked to better health outcomes and lower overall costs and that the number of family physicians in a community is associated with decreased mortality.^3^ The numbers of family physicians trained are, however, constrained by limited postgraduate educational resources available in each of those countries.

There is a relative shortage of doctors in rural areas of Africa, with understaffing of district and provincial hospitals which act as filters for referrals to specialist services in central hospitals. Most doctors working in mission and district hospitals are medical officers. They have little if any postgraduate training, but they manage a wide range of challenging medical and surgical problems with little support, supervision or backup. They are often responsible for managing district health services, including oversight of primary health care provision by nurses and other community-based cadres. They are also increasingly the educators of the next generation of health professionals through community-based education.

District-based family medicine training and specialisation could solve this mismatch of resources. The specialist family physician is a care provider, capacity builder, clinical trainer, consultant and leader of clinical governance. They see referrals from the rest of the health care team, support community-orientated primary care and work closely with the district medical officer on public health matters.^4^ Leadership from family medicine, in a strong partnership with public health, could address many national health priorities. Implementation of such community-orientated primary care could better address, for example, tuberculosis (TB) and human immunodeficiency virus (HIV) programmes, antiretroviral treatment for children and young people, prevention of maternal and neonatal mortality and morbidity, chronic and lifelong disease management, mental health services and trauma.

In the East, Central and Southern Africa (ECSA) regions, several countries (Botswana, Democratic Republic of Congo, Ethiopia, Kenya, Lesotho, Malawi, Mozambique, Tanzania, Uganda, Zambia and Zimbabwe) have embarked on training and employing family physicians at district hospitals in order to improve the quality of care in the services closest to communities. South Africa has several years’ experience of this specialist training and is working in partnership with other African countries to support their programmes.^5^ The training model, derived from the South African Family Physician specialisation programme, depends on training taking place on the distributed platform, often in rural areas, usually at district hospitals. Faculty from the university support and supervise the local family physician clinical trainers and provide an academic programme across all training sites. The College of Family Physicians in South Africa focuses on the national licensing examination and does not represent the discipline in other ways, which is the role of the South African Academy of Family Physicians (SAAFP).

Outside of South Africa, there are very limited resources available for these training programmes, especially for assessment and quality assurance of the training processes. Individual countries in the region have limited capacity to develop high-stakes assessment with sufficient validity and reliability. The implication of this is that the number of registrars is small, the quality of clinical training and workplace-based assessment is questionable and the ability of the final examination to distinguish competent from incompetent family physicians is problematic. There are few family physicians with qualifications in Health Professions Education who can design distributed learning modules and assess trainees in the range of skills they must acquire. Trainers rely heavily on materials adapted from South African universities. Other medical specialist training programmes have addressed these constraints by developing a critical mass of trainers and assessors through colleges across ECSA. We propose that a College of Family Medicine for East, Central and Southern Africa (CoFMECSA) is established along the lines of similar colleges for surgeons, anaesthetists, physicians, paediatricians and others, with reference to the model of medical specialisation through the Colleges of Medicine in South Africa.

## Colleges of health sciences

South Africa currently has integrated systems of training and certifying specialists, with university-based Master’s in Medicine (MMed) training programmes and an exit fellowship examination offered by the various Colleges of Medicine of South Africa (CMSA). This allows for the standardisation of the quality of training and assessment across the nine different medical schools in South Africa. The CMSA website states that they are the custodian of the quality of medical care in South Africa with 28 constituent colleges representing all the disciplines of medicine and dentistry within a single framework.^6^ The CMSA oversees the postgraduate specialisation of South African doctors through examination of postgraduate training in all medical specialisations. The specialists train via the MMed programme and sit the fellowship examination of the CMSA, which is recognised by the Health Professions Council of South Africa (HPCSA) for specialist registration. Therefore, family physicians specialise by completing the MMed Family Medicine programme through university medical schools, but register as specialists with the HPCSA by doing examinations run by the College of Family Physicians.

In the ECSA region (all the Southern African Development Community [SADC] countries except South Africa), the surgical associations have established a College of Surgeons (COSECSA) under the ECSA College of Health Sciences based in Tanzania. It delivers a common surgical training programme and examination and an internationally recognised surgical qualification.^7^ The curriculum is produced by the College, which sets the standards for training, with recognised training sites and trainers who conduct the training in each country. The written examination is done online. The clinical examination rotates with the College annual conference. In addition to promoting postgraduate education in surgery, COSECSA aims to advance standards of practice and research in surgical care.

The COSECSA training pathway is not only carried out at designated institutions, but also it engages hospitals and surgeons in each country that may be currently underutilised as training sites and could be configured to train. The concept of ‘colleges without walls’ is promoted, whereby surgical training is decentralised away from university teaching hospitals to accredited regional, provincial and even district hospitals. This results in greater numbers of surgical trainees staying in their home countries and providing surgical services during their training. The programme runs alongside the MMed Surgery programmes offered by the various universities of the region, and they often complement each other. Postgraduates who have trained within a university training programme are eligible to sit the membership and fellowship examinations of COSECSA, thus joining the regional College. Six different specialities are offered: (1) general surgery, (2) orthopaedics, (3) neurosurgery, (4) urology, (5) paediatrics and (6) plastic surgery. COSECSA currently operates in 14 countries in the sub-Saharan region: Burundi, Ethiopia, Kenya, Malawi, Mozambique, Namibia, South Sudan, Rwanda, Tanzania, Uganda, Zambia, Zimbabwe, Sudan and Botswana. Nearly 200 surgeons have now graduated from the COSECSA programme.

COSECSA has been successful because of the significant financial and training support it has received from international partners, including the Royal College of Surgeons in Ireland, the Royal College of Surgeons of Edinburgh, the University of Toronto, the Pan-African Academy of Christian Surgeons and the University of Oxford COSECSA Orthopaedic Link Programme, amongst others. The partnerships provide training-of-trainer courses, joint development of e-learning platforms and electronic logbook applications, support for IT labs and establishment of a database of accredited trainers and surgeons in the COSECSA region. They have an active website (www.cosecsa.org) and host the *East and Central African Journal of Surgery*.

Following the success of COSECSA, other medical disciplines have set up similar colleges for the region, with ambitions to resolve the shortage of specialists in the region through international collaborations. The ECSA College of Physicians (ECSACOP) was officially inaugurated in 2015 and has thus far held two scientific and annual general meetings in Zimbabwe (2016) and Uganda (2017). ECSACOP receives individual and institutional support from the Royal College of Physicians (London), and its Secretariat is currently housed within headquarters of the Infectious Disease Institute, Makerere campus in Kampala, Uganda. Similarly, Colleges of Obstetrics and Gynaecology (ECSACOG), Nursing (ECSACON), Ophthalmology (COECSA) and Pathology (COPECSA) have recently been established within the ECSA Health Community (www.ecsahc.org).

CANECSA is the College of Anaesthesiologists for the ECSA region, which grew out of a meeting in Zimbabwe in 2008 and 2009.^8^ Constituent member countries as of December 2014 are Kenya, Malawi, Mozambique, Rwanda, Eswatini, Tanzania, Uganda, Zambia and Zimbabwe. Although less well-resourced than the surgeons, CANECSA similarly aims to advance education and standards in safe anaesthesia and critical care in the region, to harmonise training and examinations and to standardise care. Although South Africa is not formally part of the College because it has its own college system in South Africa, the College of Anaesthesia in South Africa has supported CANECSA in the training of trainers and assessment processes. Centres with particular expertise, such as in cardiac anaesthesia, have become the focus of training for the whole region. Medical professions’ councils recognise the fellowship and CANESCA examinations as exit qualifications for registration as specialists. Every country is represented by the nominated members on the CANECSA Council, with elected positions of authority from council members with an annual general meeting of Fellows of the College.

## Proposed College of Family Medicine for East, Central and Southern Africa

Given the success of developments within other medical and nursing disciplines, it makes sense now to discuss the establishment of a College of Family Medicine for the region along similar lines. Countries that have succeeded in setting up MMeds in Family Medicine in the region have emulated the South African model, using a competency-based model, adapting its curriculum, portfolio and use of distributed learning methods and e-learning platforms. A College of Family Medicine in ECSA would be a member of WONCA Africa, as with the SAAFP. The Primary Care and Family Medicine Network for Sub-Saharan Africa (PRIMAFAMED), the international institutional network that aims to develop and strengthen family medicine higher education and training in Africa through capacity building, curricula enhancement and academic research development, would continue to support training and assessment of Family Medicine throughout the region in collaboration with CoFMECSA. Colleges of remote and rural medicine, such as in Australia and Canada, have expertise in the education of trainees who are dispersed across wide geographical areas and could partner with a regional college rather than individual associations. In addition, family physicians who are part of the African diaspora working in those countries could contribute through this single route of engagement.

Benefits and resources that could be gained through regional collaboration are listed as follows:

- offering of examinations at an international level of assessment, validity and reliability
- development of an examination question bank repository for written and oral scenarios
- enhancing the quality of training of trainers’ workshops
- use of external examiners to ensure a uniform quality of training programmes
- agreement on a single common curriculum
- collaborative research funding
- joint application for grants and research funding to benefit the region.

The next steps are for family medicine associations in each country to discuss and approve this proposal, to come together to pass a joint resolution and to submit this to the ECSA College of Health Sciences in Tanzania. Colleges such as CANECSA and COSECSA could provide guidance within countries on the process they followed.

In conclusion, the resources required to train and examine family medicine trainees to a high standard are thinly spread throughout the region, although there is high commitment to produce cadres of this discipline in many countries. We can follow the success of COSECSA, CANECSA and others by establishing a College of Family Medicine that will utilise specialist training resources more effectively, with the support and endorsement of partners such as SAAFP, PRIMAFAMED and WONCA Africa.
